# Astrocyte Inositol Triphosphate Receptor Type 2 and Cytosolic Phospholipase A_2_ Alpha Regulate Arteriole Responses in Mouse Neocortical Brain Slices

**DOI:** 10.1371/journal.pone.0042194

**Published:** 2012-08-02

**Authors:** Lihua He, David J. Linden, Adam Sapirstein

**Affiliations:** 1 Department of Anesthesiology and Critical Care Medicine, The Johns Hopkins University School of Medicine, Baltimore, Maryland, United States of America; 2 Department of Neuroscience, The Johns Hopkins University School of Medicine, Baltimore, Maryland, United States of America; University of Muenster, Germany

## Abstract

Functional hyperemia of the cerebral vascular system matches regional blood flow to the metabolic demands of the brain. One current model of neurovascular control holds that glutamate released by neurons activates group I metabotropic glutamate receptors (mGluRs) on astrocytes, resulting in the production of diffusible messengers that act to regulate smooth muscle cells surrounding cerebral arterioles. The acute mouse brain slice is an experimental system in which changes in arteriole diameter can precisely measured with light microscopy. Stimulation of the brain slice triggers specific cellular responses that can be correlated to changes in arteriole diameter. Here we used inositol trisphosphate receptor type 2 (IP_3_R2) and cytosolic phospholipase A_2_ alpha (cPLA_2_α) deficient mice to determine if astrocyte mGluR activation coupled to IP_3_R2-mediated Ca^2+^ release and subsequent cPLA_2_α activation is required for arteriole regulation. We measured changes in astrocyte cytosolic free Ca^2+^ and arteriole diameters in response to mGluR agonist or electrical field stimulation in acute neocortical mouse brain slices maintained in 95% or 20% O_2_. Astrocyte Ca^2+^ and arteriole responses to mGluR activation were absent in IP_3_R2**^−^** ^/−^ slices. Astrocyte Ca^2+^ responses to mGluR activation were unchanged by deletion of cPLA_2_α but arteriole responses to either mGluR agonist or electrical stimulation were ablated. The valence of changes in arteriole diameter (dilation/constriction) was dependent upon both stimulus and O_2_ concentration. Neuron-derived NO and activation of the group I mGluRs are required for responses to electrical stimulation. These findings indicate that an mGluR/IP_3_R2/cPLA_2_α signaling cascade in astrocytes is required to transduce neuronal glutamate release into arteriole responses.

## Introduction

Blood flow to the brain is precisely regulated to match regional perfusion with metabolic requirements. Local activation of neurons produces signals that increase regional cerebral blood flow (rCBF) in a process known as functional hyperemia. Conversely, increases in arterial perfusion pressure are countered by pressure-induced increases in myogenic tone to stabilize blood flow. Thus the cerebral vasculature is able to maintain appropriate rCBF by both vasodilation and vasoconstriction.

Recent experimental work has established a model of cerebral vascular regulation that has at its center an astrocyte-dependent signaling pathway (reviewed [1], [2]). The cytoarchitecture of astrocytes makes such a model feasible because they form a physical bridge between neural synapses and vascular structures. Astrocyte processes envelop many glutamatergic synapses and these same astrocytes also send specialized foot processes that cover the blood vessels of the brain [3], [4]. In one current model of neurovascular regulation, activation of excitatory neurons results in the presynaptic release of glutamate (and sometimes other neurotransmitters). Glutamate interacts with neuronal post-synaptic receptors but can also bind group I mGluRs of a nearby astrocyte. Early work supporting this model found that vascular responses were prevented by antagonists of group I metabotropic glutamate receptors (mGluR) and were triggered by agonist-induced activation of the mGluR [5].

Astrocyte mGluR activation is coupled to Gq and activates phospholipase C which hydrolyzes phosphatidylinositol 4,5-bisphosphate from cellular membranes to produce inositol 1,4,5-trisphosphate (IP_3_) and 1,2-diacylglycerol (DAG). IP_3_ then binds a cognate receptor, the IP_3_R, on the cytosolic face of the endoplasmic reticulum. Within astrocytes of the neocortex the only form of IP_3_R expressed is the type 2 IP_3_R (IP_3_R2). IP_3_R2 binding opens a Ca^2+^ channel within the receptor causing Ca^2+^ mobilization from internal stores [6]. Consistent with this portion of the model, direct mechanical activation of astrocytes in cortical brain slices caused arteriolar dilation which was eliminated by the cell-permeant Ca^2+^ chelator BAPTA/AM [5].

The phospholipases A_2_ (PLA_2_s) are a family of enzymes that hydrolyze a free fatty acid from the sn-2 position of membrane glycerolphospholipids and are highly expressed in the brain [7]. Previous experiments have suggested that increases in astrocyte Ca^2+^ can activate Ca^2+^-dependent PLA_2_ and that a Ca^2+^-dependent PLA_2_ is needed for cerebrovascular regulation [8]. Thus, the next step in the model is that PLA_2_ releases arachidonic acid which is metabolized by cyclooxygenase enzymes to form prostaglandin (PG) H_2_ and by epoxygenase enzymes to form epoxyeicosatrienoic acids (EETs). PGH_2_ is rapidly metabolized by terminal synthase enzymes to any of the PGs. The PGs have demonstrated vascular effects that are mediated through prostaglandin and thromboxane receptors located on the extracellular surface of vascular smooth muscle cells (VSMC). It appears that cerebral metabolism is coupled to PGE_2_-dependent vasoregulation. A low oxygen tension in brain slices increases glycolysis which produces lactate which reduces PGE_2_ uptake by astrocyte prostaglandin transporters [9]. Increased extracellular PGE_2_ dilates cerebral arterioles while cyclooxygenase inhibitors prevent vasomotor response [9], [10].

While there is some experimental evidence to support this model of neurovascular regulation, many details remain unresolved. For example, experiments supporting the present model have relied on photolysis of caged IP_3_ in astrocytes to trigger vascular responses [11]. However photo-activation of caged IP_3_ releases supraphysiologic levels of IP_3_ and does so in a way that may not represent the spatially-regulated release from subcellular compartments. In addition, the roles of PLA_2_s in neurovascular regulation have been principally examined by using pharmacologic inhibitors that are not specific for single PLA_2_ isoforms and do not target specific cell types. Mammalian brain tissue expresses, and has enzymatic activity for all of, the major PLA_2_s including Ca^2+^-independent (iPLA2, GVIA), Ca^2+^-dependent secretory PLA_2_s (groups IIA, V and X) and the cytosolic PLA_2_s (cPLA_2_, GIV) [7]. The group IVA PLA_2_ (cytosolic PLA_2_α, cPLA_2_α) is of particular interest because its translocation to specific cellular membranes is highly regulated and its enzymatic activity is enhanced by phospholipids that have arachidonate at the sn-2 position [12]. These pharmacologic inhibitors have effects on different forms of PLA_2_ and may also have side-effects that are unrelated to PLA_2_ blockade [13], [14].

Here we have sought to determine the molecular constituents of astrocyte Ca^2+^ signaling and PLA_2_ activation in the cerebrovascular regulatory pathway. To do this we compared responses of cortical astrocytes and their neighboring arterioles in cortical brain slices derived from mice that were deficient in IP_3_R2 or cPLA_2_α.

## Results

In one current model of neurovascular regulation it is postulated that activation of astrocyte mGluR by glutamate leads to activation of PLC which releases IP_3_. The free IP_3_ binds to IP_3_ receptors on the endoplasmic reticulum thus opening Ca^2+^ channels and increasing intracellular Ca^2+^ [8]. There are three IP_3_R isoforms but the type 2 receptor (IP_3_R2) appears to be the only form expressed in glial cells within the CNS [15], [16]. Therefore we measured the astrocyte Ca^2+^ and arteriole responses to stimulation of cortical brain slices from mice deficient in the type 2 IP_3_R (IP_3_R2**^−^** ^/−^) and their wild-type littermates (IP_3_R2^+/+^).

**Figure 1:**
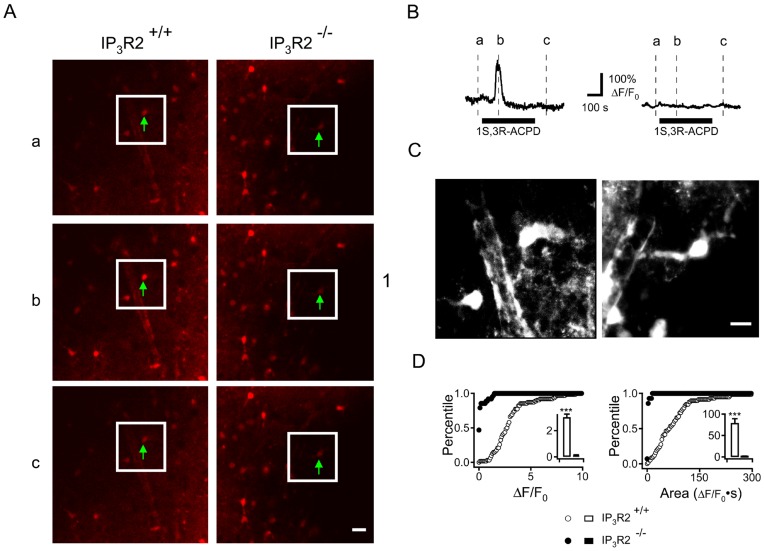
Astrocyte Ca^2+^ responses to mGluR agonist application are attenuated IP_3_R2^−/−^ slices. **A.** Neocortical brain slices from IP_3_R2^+/+^ (left panel) and IP_3_R2**^−^** ^/−^ (right panel) mice were loaded with the Ca^2+^-sensitive fluorophore Rhod-2/AM and astrocytes were identified by dye uptake, morphology and location. Ca^2+^ fluorescence was measured in the region of interest (green arrow) and is displayed at 3 time points in relation to 1S,3R-ACPD treatment: (a) before, (b) at peak response and (c) after. White outline indicates the region of magnification in C. Scale bar: 20 µm. **B.** Fluorescence intensity signals for the Ca^2+^ fluorescence measured in the soma of the indicated astrocytes. Signals were corrected for background that was measured in an identical area immediately adjacent to the region of interest. Representative single traces of the Ca^2+^ response in soma of IP_3_R2^+/+^ astrocytes (left trace) and IP_3_R2**^−^** ^/−^ (right trace) are shown and the duration of 1S, 3R-ACPD application is indicated below the traces. **C.** Z-stack of 12 images encompassing 12 mm of depth in IP_3_R2^+/+^ (left panel) and IP_3_R2**^−^** ^/−^ (right panel) brain slices. This demonstrates the ameboid shape of the astrocyte soma which extends a foot process near a neighboring arteriole. Scale Bar: 10 µm. **D.** Cumulative probability histograms of population responses are shown. Peak (left panel) and integrated (right panel) Ca^2+^ responses of IP_3_R2^+/+^ (open circles, 58 cells) and IP_3_R2**^−^** ^/−^ (filled circles, 63 cells) astrocytes with inset bar graphs indicating the mean ± S.E.M. Nine slices were prepared from four mice for each genotype.

We incubated acute cortical slices from IP_3_R2^+/+^ and IP_3_R2**^−^** ^/−^ mice with a Ca^2+^ sensitive fluorophore, Rhod-2/AM, in a manner that preferentially loads astrocytes [8]. After washout of unloaded dye and a period of equilibration in artificial CSF (ACSF) we introduced the metabotropic glutamate receptor agonist, 1S,3R-ACPD (50 µM) to the bath and examined the Ca^2+^ responses with confocal fluorescence microscopy (Figure 1A). Signals were measured in the soma of cortical astrocytes. These astrocytes were identified by their location, somatic morphology and the presence of a foot process extending from the soma (Figure 1C). In the IP_3_R2^+/+^ slices there were robust increases in the Ca^2+^ signals in cells that were morphologically identified as astrocytes, while in the IP_3_R2**^−^** ^/−^ slices such Ca^2+^ responses were absent (Figure 1B and D) (IP_3_R2^+/+^, ΔF/F_0_ = 302.4±23.5%, Time Integrated F/F_0_ = 78.8. ±10.3. IP_3_R2**^−^** ^/−^, no detectable Ca^2+^ signal. *P*<0.001. n = 58 IP_3_R2^+/+^ and 63 IP_3_R2**^−^** ^/−^ cells from 4 mice for each genotype).

**Figure 2:**
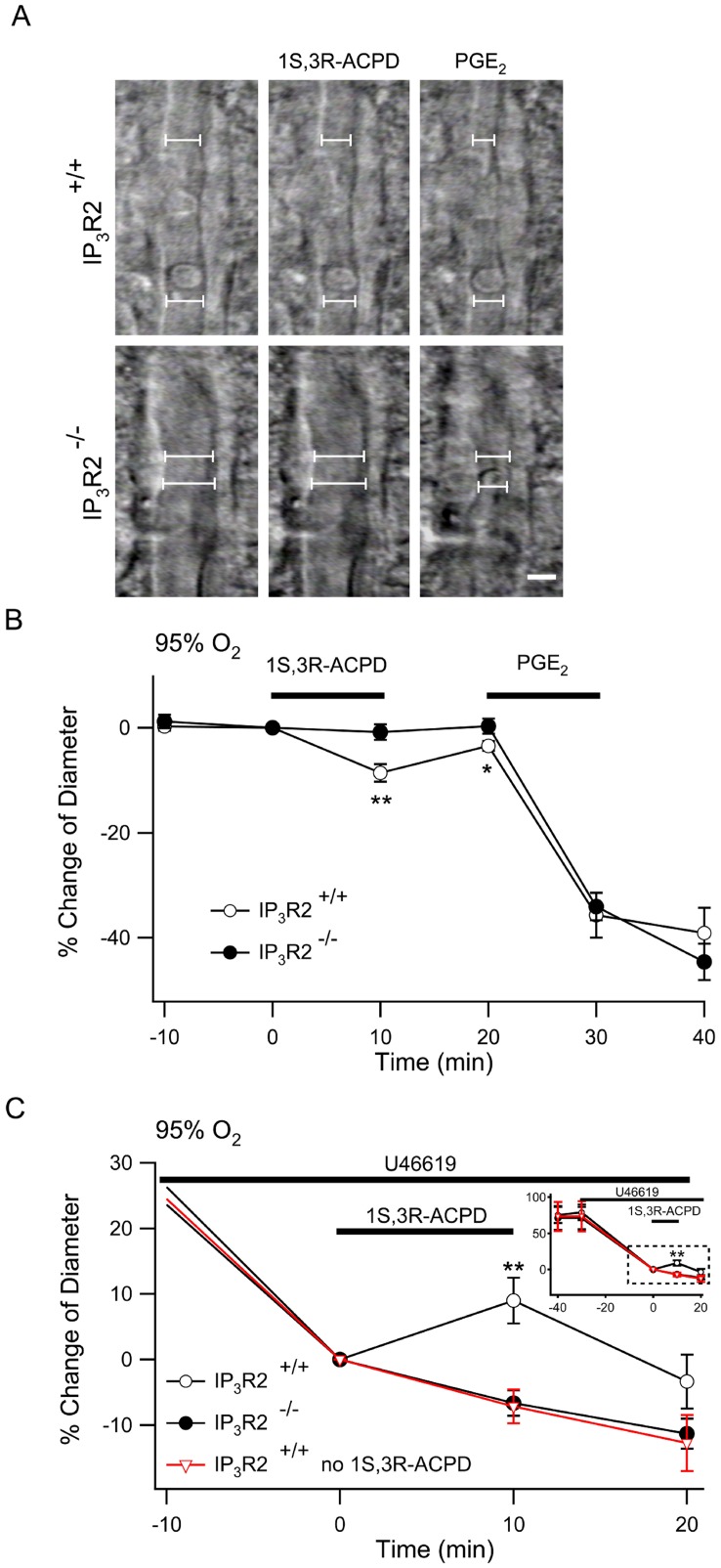
Arteriole responses to mGluR agonist application are eliminated in IP_3_R2^−/−^ neocortical slices. **A.** Gradient contrast imaging was used to measure arteriole responses to treatment. Responses to 1S,3R-ACPD were quantified by defining 6 points (2 of the points are shown in this figure) at which to measure changes in arteriole diameter over time (described in Methods). The arteriole diameter is expressed as the average diameter of the points. Scale bar: 5 µm. **B.** Cortical slices from IP_3_R2^+/+^ (open circle, n = 18) and IP_3_R2**^−^** ^/−^ (filled circle, n = 24) were treated with 1S,3R-ACPD followed by PGE_2_ and arteriole responses were measured. **C.** During continuous application of U-46619 the IP_3_R2^+/+^ and IP_3_R2**^−^** ^/−^ were treated with 1S,3R-ACPD while another group of IP_3_R2^+/+^ slices were treated with vehicle instead of 1S,3R-ACPD (red inverted triangle, n = 7). Inset shows the complete experiment from the time of application of U-46619. The dashed white box indicates the expanded graph. Treatment with U-46619 (100 nM) constricted arterioles of both IP_3_R2^+/+^ (n = 16) and IP_3_R2**^−^** ^/−^ slices (n = 17) to a similar extent. **, *P*<0.01 comparing IP_3_R2^+/+^ to IP_3_R2**^−^** ^/−^10 min following 1S, 3R-ACPD application.

We measured changes in the diameter of arterioles in cortical brain slices from IP_3_R2^+/+^ and IP_3_R2**^−^** ^/−^ mice in response to 1S,3R-ACPD (Figure 2). Populations of arterioles were selected as described in Methods so that the mean diameters before any treatment were similar in the IP_3_R2^+/+^ and IP_3_R2**^−^** ^/−^ slices. After equilibration in ACSF with 95% O_2_ and 5% CO_2_ the diameters of IP_3_R2^+/+^ (n = 18) and IP_3_R2**^−^** ^/−^ arterioles (n = 24) were 11.8±1.1 µm and 10.1±0.8 µm respectively (*P*  = 0.20). In the 95% O_2_ environment, treatment of IP_3_R2^+/+^ slices with 1S,3R-ACPD caused significant arteriole constriction while the IP_3_R2**^−^** ^/−^ arterioles did not respond (Figure 2A, B) (IP_3_R2^+/+^, 8.8±1.7%, *vs.* IP_3_R2**^−^** ^/−^, 0.8±1.5%; *P*<0.01). Prostaglandin E_2_ (PGE_2_) is a vasoactive metabolite of arachidonic acid that is thought to participate in vascular regulation through activation of prostaglandin E receptors (reviewed in [17]). Arterioles in IP_3_R2^+/+^ and IP_3_R2**^−^** ^/−^ mice had the same constrictive response to treatment with PGE_2_ (IP_3_R2^+/+^, −35.7±4.3% *vs.* IP_3_R2**^−^** ^/−^, −34.1±2.6%; *P* = 0.74) which indicates the mechanisms for prostaglandin-dependent vasoregulation are downstream from astrocyte IP_3_ signaling and that arterioles in IP_3_R2**^−^** ^/−^ mice are not generally deficient in constrictive function. Other investigators have suggested that the pre-existing level of vascular tone (diameter) determines the vasomotor response to mGluR activation [10] and have postulated that vessels without myogenic tone, as is the case in brain slices, do not represent a physiologic state [2]. In order to simulate physiologic levels of arteriolar tone we bath-applied U-46619, a stable analog of PGH_2_, that is a selective thromboxane receptor agonist [18]. U-46619 (1 µM) caused complete occlusion of many arterioles while lower doses resulted in continuous arteriole constriction with a slope that was dose-dependent (not shown). We applied 100 nM U-46619 because it constricted the arterioles approximately 40% of the initial diameter within the equilibration period of the experiment (Supporting Figure S1). When cortical slices from IP_3_R2^+/+^ and IP_3_R2**^−^** ^/−^ slices were pretreated with 100 nM U-46619 they constricted to the same degree (Constriction relative to diameter after 30 minutes of U-46619: IP_3_R2^+/+^, −78.9±10.6%, n = 16 vs. IP_3_R2**^−^** ^/−^, −70.9±15.4%, n = 17; *P*  = 0.29) (Figure 2C, inset). Supporting Figure S2 shows a representative trace of diameter from a single arteriole. Bath application of 1S,3R-ACPD (50 µM for 10 min) during U-46619 exposure caused significant IP_3_R2+/+ arteriole dilation (16.1±3.5%, *P*<.01) while there was no response in the IP_3_R2**^−^** ^/−^ arterioles (0.5±2.0%) when compared to IP_3_R2+/+ slices that were not treated with 1S,3R-ACPD (n = 7) (Figure 2C).

**Figure 3:**
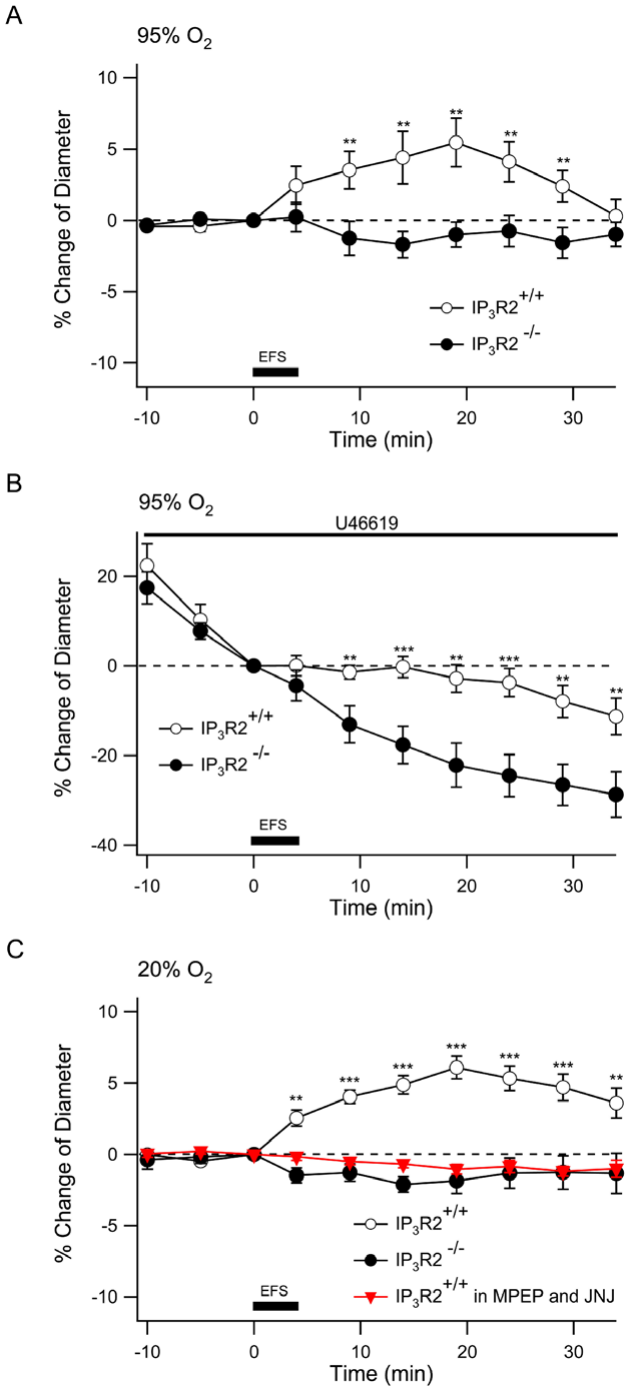
Neocortical arterioles of IP_3_R2^−^^/−^ slices do not respond to electrical field stimulation. **A.** Responses of arterioles in 95% O_2_ following electrical field stimulation. Cortical slices from IP_3_R2^+/+^ (open circle) and IP_3_R2**^−^** ^/−^ mice (closed circle) were treated with electrical field stimulation (EFS) of 100 Hz trains of 200 ms at 0.2 Hz for 4 minutes as indicated by the dark bar. Arteriole diameter was measured every 5 min during the experiment. IP_3_R2^+/+^, n = 15; IP_3_R2**^−^** ^/−^ n = 15. **, *P*<0.01, IP_3_R2^+/+^ compared to IP_3_R2**^−^** ^/−^. **B.** Pretreatment with U-46619 for 30 min was followed by electrical stimulation and arteriole diameters of IP_3_R2**^−^** ^/−^ were compared to IP_3_R2^+/+^. IP_3_R2^+/+^, n = 15; IP_3_R2**^−^** ^/−^ n = 14. **, *P*<0.01; ***, *P*<0.001. **C.** Blockade of Group I mGluR with MPEP and JNJ prevents arteriole responses to electrical stimulation in IP_3_R2^+/+^ slices (inverted triangles) while a 20% O2 environment does not alter responses of naïve IP_3_R2^+/+^ or IP_3_R2**^−^** ^/−^ arterioles. IP_3_R2^+/+^, n = 14; IP_3_R2**^−^** ^/−^ n = 12, IP_3_R2^+/+^ with MPEP/JNJ, n = 9. **, *P*<0.01; ***, *P*<0.001.

Treatment with 1S,3R-ACPD initiates the vasoregulatory pathway at the point of astrocyte mGluR activation and thus bypasses upstream signaling in neurons. In order to examine the role of the astrocyte IP_3_ signaling on neurovascular coupling following activation of neurons we performed electrical field stimulation in the cortical brain slices. We stimulated a cortical field with a bipolar electrode using 100 Hz for 200 ms repeated 48 times over a 4 min period and examined the arteriolar responses 200–300 µm from the electrode. We determined that this 100 Hz stimulation increased astrocyte somatic intracellular Ca^2+^ consistent with their robust activation (Supporting Figure S3). In contrast to treatment with 1S,3R-ACPD, electrical field stimulation caused significant dilation in IP_3_R2^+/+^ arterioles, which peaked 15 min after stimulation (5.45±1.7%, n = 15). There was an insignificant constriction of 1.70±0.92% in the IP_3_R2**^−^** ^/−^ slices (Figure 3A) (n = 14; *P*<0.01 compared to IP_3_R2^+/+^ between 5–30 minutes following stimulation). When the slices were pre-treated with U-46619 (50 nM), electrical stimulation caused a significant dilatory response in the IP_3_R2^+/+^ arterioles when compared to the continuous constriction observed in the IP_3_R2**^−^** ^/−^ slices (Figure 3B) (IP_3_R2^+/+^, −2.8±3.1% vs. IP_3_R2**^−^** ^/−^, −22.2±5.0%; measured 15 minutes after electrical stimulation, expressed relative to diameter at start of stimulation; *P*<0.01).

In contrast to our results, a previous study in rat hippocampal slices found that electrical stimulation in a high O_2_ environment caused arteriole constriction [9]. However, in a low O_2_ environment, electrical stimulation caused arteriole dilation [9]. Therefore we wished to determine if O_2_ tension alters the response to electrical stimulation of murine cortical slices. When electrical stimulation was delivered to IP_3_R2^+/+^ and IP_3_R2**^−^** ^/−^ slices equilibrated in 20% O_2_ the responses were identical to those observed in 95% O_2_ (Figure 3C, compare to 3A). To determine if electrical stimulation of neurons causes dilation by activation of the astrocyte mGluR, slices from IP_3_R2^+/+^ were treated with the mGlu_5_ antagonist, 2-Methyl-6-(phenylethynyl)pyridine hydrochloride (MPEP) (10 µM) and the mGlu_1_ antagonist JNJ 16259685 (100 nM) before electrical stimulation. Blockade of these group I mGluRs prevented the arteriole dilation response to stimulation (Figure 3C). Taken together these results show that, glutamatergic activation of astrocyte mGluRs results in IP_3_R2 receptor-mediated increases in astrocyte Ca^2+^ that are necessary for either constriction or dilation of arterioles. The direction of the change in arteriole diameter depends on the pre-existing state of the vessel (U-46619 pre-treatment) and also the mode of activation (1S, 3R-ACPD vs. electrical stimulation).

**Figure 4:**
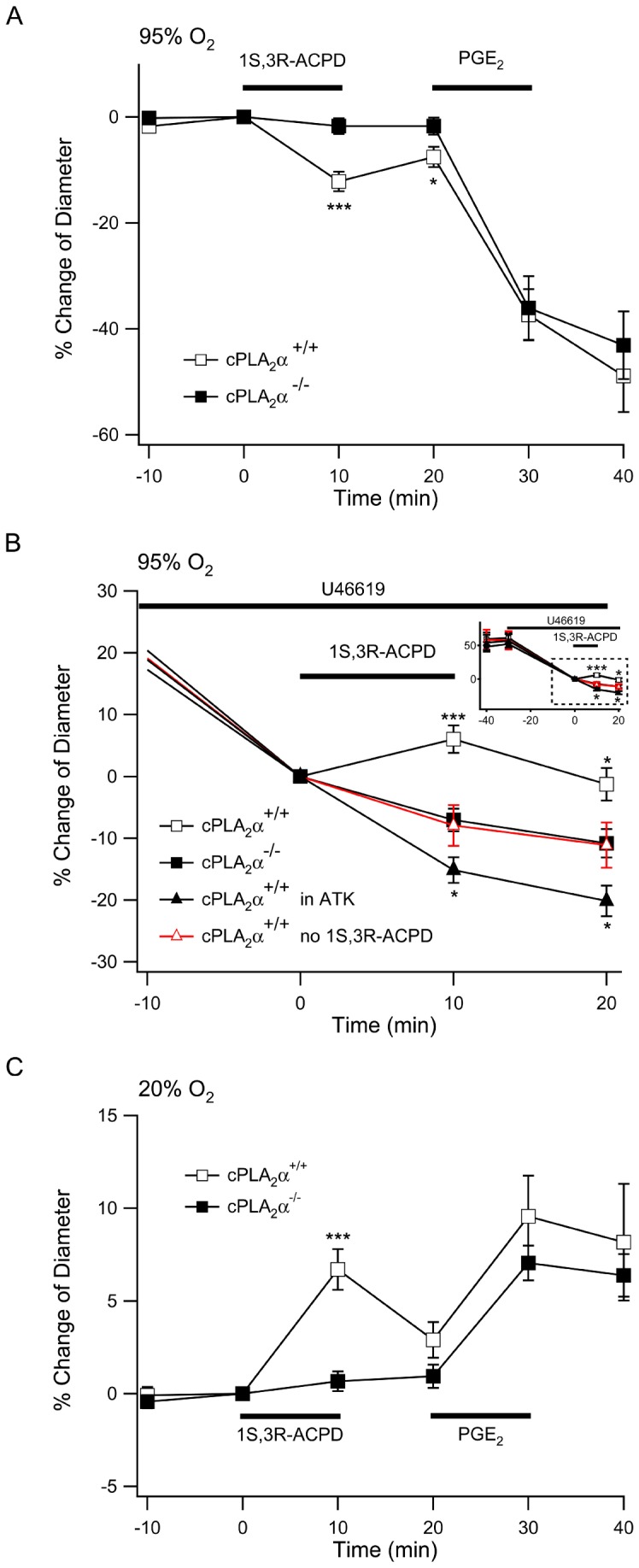
Arteriole responses to mGluR agonist application are eliminated in cPLA_2_α^−/−^ neocortical slices. **A.** Bath application of 1S,3R-ACPD 50 µM to slices equilibrated with 95% O_2_ induced constriction of arterioles in cortical slices of cPLA_2_α^+/+^ (empty square, n = 29), but not cPLA_2_α **^−^** ^/−^ mice (filled square, n = 15). Washout of 1S,3R-ACPD was followed by application of 10 µM PGE_2_ which caused identical constriction of arterioles in both genotypes. **B.** In slices at equilibrium with 95% O_2_, arterioles were preconstricted with 100 nM U-46619 supplemented ACSF. To compare acute cPLA_2_α inhibition with gene deletion, cPLA_2_α^+/+^ slices were treated with 10 µM ATK (filled black triangle, n = 21) for the duration of the experiment. Inset shows the complete experiment from the time of application of U-46619. The dashed white box indicates the expanded graph. After 30 min equilibration in U-46619, 1S,3R-ACPD was added to the bath at a final concentration of 50 µM (time  = 0) and the responses of arterioles in cPLA_2_α^+/+^ (n = 21) and cPLA_2_α**^−^** ^/−^ (n = 25) cortical slices were compared to cPLA_2_α^+/+^ slices that were not treated with 1S,3R-ACPD (red empty triangle, n = 10). *, *P*<0.05; **, *P*<0.01; ***, *P*<0.001. **C.** When slices were equilibrated in 20% O_2_, 1S,3R-ACPD treatment dilated cPLA_2_α^+/+^ (n = 18) but not cPLA_2_α**^−^** ^/−^ (n = 16) arterioles. Bath application of 10 µM PGE_2_ caused dilation of both genotypes. ***, *P*<0.001.

Increases in astrocyte Ca^2+^ are postulated to activate one or more forms of Ca^2+^-dependent PLA_2_ and PLA_2_ activity may be the rate limiting step in the generation of vasoactive eicosanoids [19]. Because of the unique biochemical and molecular properties of cPLA_2_α we explored arteriole responses in acute cortical brain slices derived from cPLA_2_α^+/+^ and cPLA_2_α**^−^** ^/−^ mice. Once again we selected arterioles based on the histology and diameter of the vessel and determined that there was no difference in the arteriole diameters between cPLA_2_α^+/+^ (11.6±1.3 µm, n = 29) and cPLA_2_α**^−^** ^/−^ neocortical slices (11.8±1.1 µm, n = 15). We repeated the 1S,3R-ACPD stimulation experiment in a 95% O_2_ environment and found that in naïve cPLA_2_α^+/+^ slices, arterioles constricted 12.2±1.9% in response to 1S,3R-ACPD. In contrast arterioles in slices of cPLA_2_α**^−^** ^/−^ neocortex did not constrict in response to 1S, 3R-ACPD (1.6±1.3%; *P*<.001 compared to cPLA_2_α^+/+^, Figure 4A). We postulated that cPLA_2_α serves to supply arachidonic acid and predicted that the response to exogenous PGE2 would be unaltered in cPLA_2_α**^−^** ^/−^ slices [5]. When 1S, 3R-ACPD was removed from the perfusate and replaced with 10 µM PGE2, arterioles of both the cPLA2α+/+ and cPLA_2_α**^−^** ^/−^ slices constricted identically (cPLA2α+/+, 35.1±5.2%; cPLA_2_α**^−^** ^/−^, 34.4±6.6%; Figure 4A).

To determine if cPLA_2_α-dependent responses were affected by the tension of the arteriole prior to activation we pre-treated slices with U-46619. Following 30 minutes of U-46619 exposure the degree of constriction in cPLA_2_α^+/+^ (35.1±2.9%, n = 21) and cPLA_2_α**^−^** ^/−^ arterioles (31.6±2.4%, n = 25) was not different (Figure 4B). In the presence of U-46619, cPLA_2_α^+/+^ arterioles continued to progressively constrict and were used as a control for drug treatment experiments (n = 10). We applied 1S,3R-ACPD 50 µM which caused a significant 13.9±2.2% dilation in cPLA_2_α^+/+^ arterioles when compared to slices that were treated with vehicle instead of 1S,3R-ACPD (n = 10) (Figure 4B; normalized to U-46619 preconstricted arteriole diameters immediately before treatment with 1S,3R-ACPD or vehicle; *P*<.01, 15 min following electrical stimulation). In contrast, the arterioles of cPLA_2_α**^−^** ^/−^ slices did not respond to 1S,3R-ACPD treatment (0.9±1.8%). To ensure that the results measured in the cPLA_2_α**^−^** ^/−^ slices were due to loss of enzymatic activity and not an unrelated mechanism compensating for gene deletion, we pretreated cPLA_2_α^+/+^ slices with 10 µM ATK (n = 21), a mixed iPLA_2_/cPLA_2_α inhibitor, during the 30 min U-46619 preconstriction. ATK treatment prevented dilation in the 1S,3R-ACPD-treated cPLA_2_α^+/+^ arterioles and resulted in a small constrictive response to 1S,3R-ACPD when compared to slices that were treated with U-46619 but not 1S,3R-ACPD (ATK/U-46619/1S,3R-ACPD-treated −15.2±2.1%, compared to U-46619-treated −7.9±3.3%; *P*<0.05).

In a previous investigation the polarity of arteriole response to t-ACPD was dependent upon oxygen content of the slice media [9]. A low oxygen environment appeared to enhance glycolysis with increased lactate and PGE_2_ levels and resulted in dilation of arterioles [9]. Therefore we equilibrated slices from cPLA_2_α^+/+^ and cPLA_2_α**^−^** ^/−^ mice in 20% O_2_ and 5% CO_2_ before and during treatment with 1S, 3R-ACPD. In low O_2_, pharmacological activation of the mGluR resulted in dilation of the cPLA_2_α^+/+^ arterioles while cPLA_2_α**^−^** ^/−^ arterioles remained unresponsive (Figure 4C). Interestingly the 20% O_2_ environment also reversed the polarity of the response to 10 µM PGE_2_, causing arteriole dilation in both genotypes (Figure 4C). Taken together these results show that cPLA_2_α is required for the vascular responses to 1S,3R-ACPD stimulation of the mGluR. Importantly, cPLA_2_α reaction products and their metabolites can trigger either arteriole constriction or dilation, depending on the initial condition and the metabolic state of the vessel.

**Figure 5:**
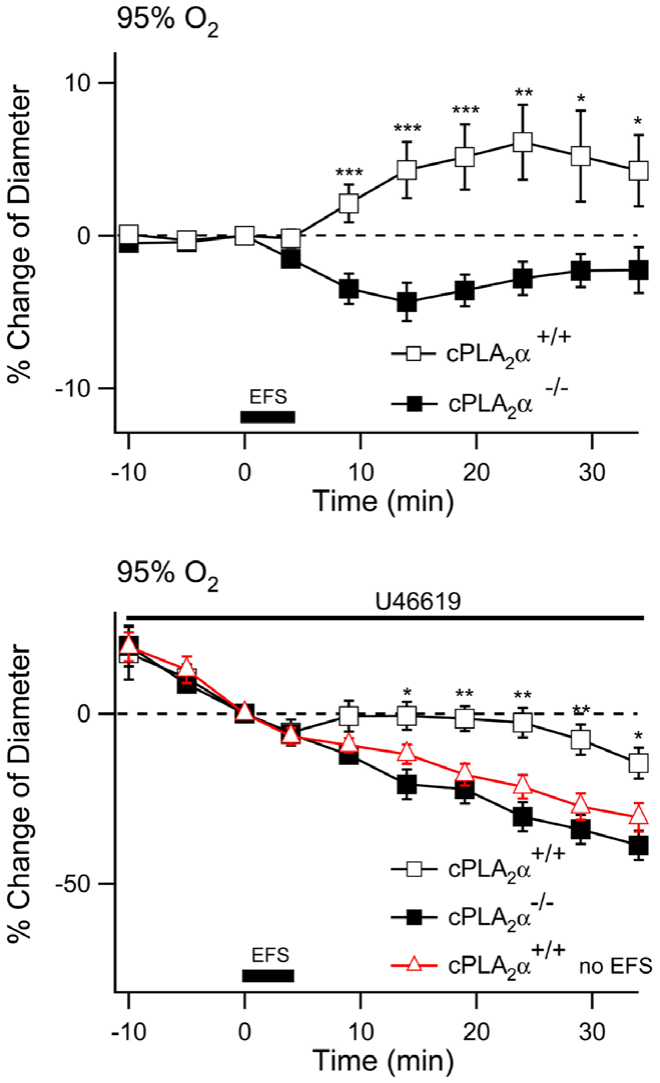
Arterioles of cPLA_2_α^−/−^ neocortical slices do not dilate in response to electrical stimulation. **A.** Neocortical brain slices from cPLA_2_α^+/+^ (empty square, n = 23) and cPLA_2_α**^−^** ^/−^ mice (filled square, n = 23) were stimulated with 100 Hz trains of 200 ms at 0.2 Hz for 4 minutes as indicated by the dark bar (electrical field stimulation, EFS). Arteriole diameter was measured every 5 min during the experiment. *, *P*<0.05; **, *P*<0.01; *** *P*<0.001: cPLA_2_α^+/+^ compared to cPLA_2_α**^−^** ^/−^. **B.** After treatment with 20 nM U-46619 for 30 min, electrical stimulation was applied to cortical slices from cPLA_2_^+/+^ and cPLA_2_α**^−^** ^/−^ mice and changes in arteriole diameter were compared to cPLA_2_α^+/+^ arterioles that did not have electrical stimulation (red triangle, n = 9). *, *P*<0.05; **, *P*<0.01; *** *P*<0.001: cPLA_2_α^+/+^ and cPLA_2_α**^−^** ^/−^ with electrical stimulation compared to cPLA_2_α^+/+^ without electrical stimulation at the same time points.

If increased astrocyte Ca^2+^ is directly coupled to activation of cPLA_2_α then we predicted that cPLA_2_α would be necessary for the arteriole response to electrical stimulation in cortical brain slices. We subjected cortical slices, in 95% O_2_, from cPLA_2_α+/+ and cPLA_2_α**^−^** ^/−^ mice to the same electrical stimulation protocol used previously. The cPLA_2_α+/+ arterioles had a small but significant dilation while the cPLA_2_α**^−^** ^/−^ arterioles constricted slightly in response to the electrical stimulation (Figure 5A) (20 min after stimulation, cPLA_2_α+/+, 6.1±2.5%, n = 23 compared to cPLA_2_α**^−^** ^/−^, −2.8±1.1%, n = 23, *P*<0.01). To determine if the vascular tone of the arteriole impacts the response to electrical stimulation we applied a lower dose of U-46619, 20 nM, to the slice perfusate. This treatment caused arteriole constriction that was the same in cPLA_2_α+/+ and cPLA_2_α**^−^** ^/−^ slices (Figure 5B). When electrical stimulation was applied to U-46619-treated cPLA_2_α+/+ slices there was a highly significant dilation response when compared to arterioles that were treated with U-46619 but not electrically stimulated (Figure 5B). This response became significant 10 min following electrical stimulation and continued through the 30 minute measurement period. In contrast there was no response of the cPLA_2_α**^−^** ^/−^ arterioles to electrical stimulation when compared to unstimulated slices (Figure 5B) (20 minutes after stimulation, cPLA_2_α+/+, −2.6±4.3%, n = 23; cPLA_2_α**^−^** ^/−^, −30.3±4.3%, n = 23; and cPLA_2_α+/+, without electrical stimulation, −21.5±3.5%, n = 9; all diameters relative to the diameter at time of the onset of electrical stimulation. *P*<0.01 for cPLA_2_α^+/+^ compared to cPLA_2_α^+/+^ without simulation). The results thus far show that 1S,3R-ACPD (isolated mGluR activation) causes constriction in naïve cPLA_2_α+/+ slices while electrical stimulation (which includes neuron activation) causes dilation. Neurons must therefore modulate vascular responses by trans-cellular activation of the astrocyte mGluR and a second mGluR-independent mechanism. This mGluR-independent mechanism must also be cPLA_2_α-dependent since arterioles in cPLA_2_α**^−^** ^/−^ slices also failed to respond to electrical stimulation.

**Figure 6:**
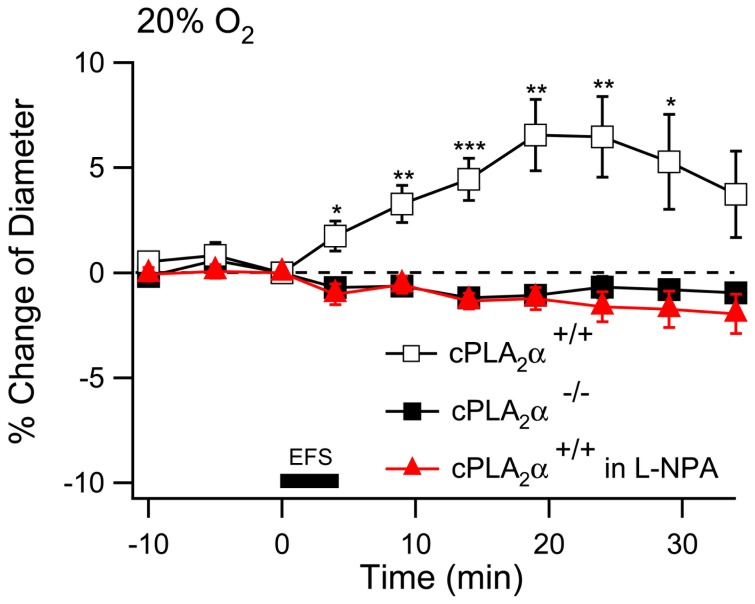
Inhibition of nNOS with L-NPA prevents dilation of arterioles following electrical stimulation. cPLA_2_α^+/+^ slices were equilibrated in 20% O_2_ and treated with ACSF (hollow square, n = 14; ) or ACSF with 10 µM L-NPA 60 min before electrical stimulation (filled red triangle, n = 9). cPLA_2_α**^−^** ^/−^ (filled square, n = 9) were treated with ACSF. Electrical stimulation was applied as indicated by the dark bar (electrical field stimulation, EFS). *, *P*<.05; **, *P*<.01.

We wished to understand the role of cPLA_2_α in arteriole regulation following electrical stimulation. Neuronal nitric oxide synthase (nNOS) is thought to be the only isoform that contributes to metabolic hyperemia [20]. In addition, neuronal nitric oxide (NO) has been postulated to modulate cerebrovascular responses by inhibition of arachidonic acid metabolism [21]. Therefore, we applied either 10 µM Nω-propyl-l-arginine (L-NPA), a specific nNOS antagonist, or vehicle to cPLA_2_α^+/+^ slices equilibrated in 20% O_2_. Slices that were treated with vehicle dilated in response to electrical stimulation just as they had in 95% O_2_ (Figure 6). In contrast L-NPA-treated and cPLA_2_α**^−^** ^/−^ slices had a small constrictive response (Figure 6). Thus it appears that nNO plays a role in the generation of the cPLA_2_α-dependent production of a vasodilator compound.

**Figure 7:**
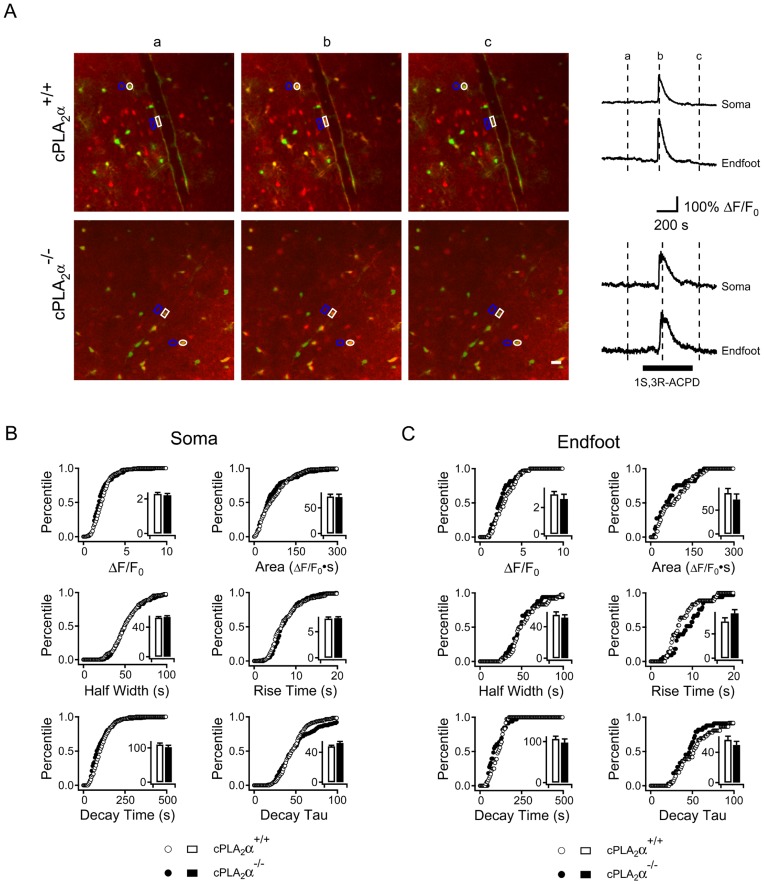
Astrocyte Ca^2+^ responses in neocortical slices to mGluR agonist application are not altered by absence of cPLA_2_α expression. Cortical brain slices from S100β-EGFP/cPLA_2_α^+/+^ (upper panel) and S100β-EGFP/cPLA_2_α**^−^** ^/−^ (lower panel) mice were loaded with the Ca^2+^-sensitive fluorophore Rhod-2/AM. Regions of interest representing astrocyte soma (white circles) and vascular foot processes (white box) were identified by EGFP expression and histologic location. Regions of interest representing background fluorescence for soma and endfeet are depicted by blue outlines. Ca^2+^ fluorescence measured for the soma and endfeet are displayed at 3 times in relation to 1S,3R-ACPD treatment: (a) before, (b) at peak response and (c) after. Representative Ca^2+^ measurements for soma and endfeet for each genotype are depicted in the right panel. The time of the 1S, 3R ACPD application is indicated by the black bar. The Ca^2+^ responses of astrocyte populations are shown in **B.** Soma (+/+, n = 169; **^−^** ^/−^, n = 166) and **C.** endfeet (+/+, n = 36; **^−^** ^/−^, n = 33) and were measured as Ca^2+^ peak amplitude, area under curve, half width, rise time, decay time or decay tau (as defined in Methods). The graphs show cumulative probability histograms analysis of the astrocyte populations by parameters compared between cPLA_2_α^+/+^ (open circles and bars) and **^−^** ^/−^ (closed circles and bars) while inset bar graph shows the mean ± S.E.M. for each parameter. There were no significant differences between the genotypes.

It is possible that cPLA_2_α is necessary for normal Ca^2+^ responses to mGluR activation. In order to precisely determine if cPLA_2_α alters the astrocyte Ca^2+^ response to 1S,3R-ACPD we crossed the cPLA_2_α gene deficiency into a mouse line carrying a transgene that expresses the EGFP under the control of the S100β promoter [22]. These mice have a population of astrocytes that strongly express EGFP which can readily be identified by fluorescent microscopy. We prepared acute cortical slices from S100β-EGFP-cPLA_2_α+/+ and S100β-EGFP-cPLA_2_α**^−^** ^/−^ mice and loaded the slices with Rhod-2/AM. Using EGFP fluorescence we identified astrocytes and defined regions of interest (ROI) around the soma and neighboring foot processes (Figure 7A). We measured the Ca^2+^ responses over time in the soma and foot processes of these cells following bath application of 1S,3R-ACPD 50 µM (Figure 7A). There were no differences between the cPLA_2_α+/+ and cPLA_2_α**^−^** ^/−^ genotypes in the Ca^2+^ responses as measured by amplitude, rise time, half width duration, decay time or total integrated signal in the soma (Figure 7B) (cPLA_2_α+/+, n = 169 cells; cPLA_2_α**^−^** ^/−^, n = 166 cells) or the foot processes (Figure 7C) (cPLA_2_α+/+, n = 36 endfeet; cPLA_2_α**^−^** ^/−^, n = 33 endfeet).

## Discussion

We have used mutant mice to identify IP_3_R2 and cPLA_2_α as essential elements for the transduction of neuronal activity into vascular responses in an acute neocortical brain slice model. We found that IP_3_R2 and cPLA_2_α are necessary for both constrictive and dilatory responses following activation of the astrocyte mGluR by 1S,3R-ACPD. Furthermore electrical stimulation of brain slices caused arteriole dilation which was dependent on IP_3_R2, cPLA_2_α and neuron-derived NO. The increase in astrocyte intracellular Ca^2+^ following application of 1S,3R-ACPD requires IP_3_R2 but not cPLA_2_α. These results are the first demonstration that the effector of mGluR-evoked Ca^2+^ response in astrocytes is the IP3R2 and that this receptor and cPLA_2_α are essential for cortical cerebrovascular regulation.

Many of the biochemical steps involved in the neuron-to-astrocyte-to-vascular smooth muscle cell signal transduction process have been defined. The sufficiency of astrocyte Ca^2+^ transients to initiate arteriole responses in brain slices was demonstrated by photo-uncaging of Ca^2+^ within astrocytes [8] and photolysis of caged IP_3_ in retinal Mueller cells which triggered both a Ca^2+^ response in the Mueller cell and vasodilation [11]. Thus, previous work is supportive of the model but photolysis may have had effects on cells other than the targeted astrocytes. Previously, use of IP_3_R2 knockout mice demonstrated that IP_3_R2 is required for the Ca^2+^ responses of hippocampal astrocytes to a combination of chemical G-protein coupled receptor activators [15]. Because IP_3_R2 is the only IP_3_R isoform that is expressed in astrocytes [15], we reasoned that if astrocyte IP_3_ is an essential effector of vascular responses to astrocyte mGluR activation then the signal must be transduced through the IP_3_R2. This reasoning is supported by the finding that IP_3_R2**^−^** ^/−^ cortical slices failed to respond to mGluR stimulation (Figures 1−2 3). Furthermore pharmacologic blockade of group I mGluRs prevented vascular responses to electrical stimulation in IP_3_R2^+/+^ slices and had no effect on IP_3_R2**^−^** ^/−^ slices (Figure 3C). IP_3_R2 may also be expressed in cerebrovascular endothelial cells and it is possible that endothelial IP_3_R2 contributes to the arteriole response to mGluR stimulation [23]. However, it is unlikely that results in the IP_3_R2**^−^** ^/−^ slices can be attributed to loss of endothelial IP_3_R2 because both blockade of mGluRs in IP_3_R2^+/+^ slices and cPLA_2_α deficiency eliminated the responses to electrical stimulation (Figures 3C and 5). The role of endothelial IP_3_R2 in vascular responses can be explicitly tested using endothelial denuded cerebral arterioles in future studies [24]. These results also demonstrate that the diacylglycerol produced by PLC activation is not sufficient to regulate arteriole responses.

Previous experiments have used non-specific inhibitors of various PLA_2_s to suggest that astrocyte Ca^2+^ signaling activates PLA_2_ and that this activity is required for cerebrovascular regulation [8], [10]. For example inhibition of PLA_2_ in intact mice by application of the drug MAFP to the cortical surface eliminated the response to photo-uncaging of Ca^2+^ in astrocytes [25]. However, the particular molecular form(s) of PLA_2_ needed for cerebrovascular regulation had not previously been determined. cPLA_2_α has been identified in astrocytes but other forms of PLA_2_ are also expressed in astrocytes [26], [27]. Our experiments show that the cPLA_2_α is the molecular species that is downstream from IP_3_R2 activation. We also examined the effect of cPLA_2_α expression on the astrocyte Ca^2+^ response following 1S,3R-ACPD treatment. The results of the present Ca^2+^ imaging experiments show that cPLA_2_α does not alter the IP_3_-mediated Ca^2+^ response or impact Ca^2+^ homeostasis in the astrocyte following efflux of Ca^2+^ from the endoplasmic reticulum in the astrocyte.

cPLA_2_α activity is regulated by phosphorylation and translocation to its membrane substrate. Translocation of cPLA_2_α to cellular membranes requires Ca^2+^ binding to the C2 domain of the protein [28] while enzymatic activity is modulated by phosphorylation at sites that are not part of the C2 domain [29]. The local cellular Ca^2+^ levels, lipid environment and the phosphorylation state of the protein play a role in determining the specific membrane compartment to which cPLA_2_α translocates [30]. When astrocyte Ca^2+^ waves were initiated in brain slices by selective photo-uncaging of astrocyte Ca^2+^, peaks in endfoot Ca^2+^ preceded those in astrocyte soma [8]. Our results demonstrate that mGluR activation causes Ca^2+^ increases in the soma and the foot processes of astrocytes. Local elevation of endfoot Ca^2+^ makes it possible that cPLA_2_α translocates to the cytosolic face of these membranes to hydrolyze arachidonic acid at this site. Localization of cPLA_2_α to the astrocyte perivascular endfoot could lead to increases in arachidonic acid concentration at the endfoot where it can be metabolized intracellularly or diffuse transcellularly into the neighboring vascular smooth muscle cell [8]. The membrane site of cPLA_2_α activity has the potential to profoundly impact the eicosanoids that are generated from arachidonic acid metabolism.

Our results are consistent with a model in which astrocyte cPLA_2_α generates the arachidonic acid that is metabolized within the astrocyte for production of prostaglandins and vasodilatory EETs. Astrocytes express COX-1 which metabolizes arachidonic acid to PGH_2_ which is the precursor for all prostaglandins [25]. PGs, particularly PGE_2_, are implicated in the arteriole dilation response [9]. Cultured cortical astrocytes contain CYP450 epoxygenase activity that metabolizes arachidonic acid to vasodilatory EETs which can regulate cerebral blood flow by transcellular passage from the astrocyte foot process to the VSMC [31], [32]. Cerebral vascular smooth muscle cells express CYP450 ω–hydroxylase which metabolizes arachidonic acid to form 20-HETE [33]. The production of 20-HETE inhibits BK channels while increasing open probability of the L-type Ca^2+^ channels of the VSMC, leading to VSMC and arteriole constriction [34]. When cPLA_2_α translocates to the vascular foot process arachidonic acid can diffuse into the VSMC where it is metabolized to vasconstrictive 20-HETE. In agreement with our results on 1S,3R-ACPD-treated, naïve brain slices, a non-specific PLA_2_ inhibitor prevented the constrictive response following uncaging of Ca^2+^ in mouse cortical brain slices [8]. Also consistent with our findings, treatment of rat brain slices with the combination of the thromboxane receptor agonist U-46619 and the non-specific PLA_2_ inhibitor methyl arachidonyl fluorophosphanate (MAFP) significantly reduced the arteriole response to 1S,3R-ACPD treatment [10].

If the roles of IP_3_ and cPLA_2_α in cerebrovascular regulation are simply to regulate the release of arachidonic acid in astrocytes for metabolism into both eicosanoid vasodilators and vasoconstrictors, what determines the response of the vessel? Our results provide some new insight into this process.

The initial diameter of an arteriole is determined by the balance between signals for constriction and dilation on the VSMC. Previous studies conducted in 95% O_2_ with rat brain slices suggested that the initial arteriole diameter determines the directionality and magnitude of changes in diameter following stimulation [10]. Arterioles in naïve slices are not pressurized and therefore lack intrinsic tone and are relatively dilated. Therefore, an arteriole in a naïve slice may be more responsive to constrictive stimuli [8], [35]. In contrast, pretreatment with U-46619 causes vasoconstriction that favors dilation following activation of the astrocyte [10]. As we observed, absence of the IP_3_R2-cPLA_2_α pathway abolishes the vasomotor effects of mGluR activation regardless of the resting diameter of the arteriole. This indicates that this signaling pathway is required for elaboration of both vasoconstrictors and dilators.

The balance of the metabolism of arachidonic acid between CYP450 and cyclooxygenase enzymes appears to be essential for neurovascular regulation [36], [37]. Post-synaptic neurons also release NO and NO has been implicated in determining the metabolic balance between synthesis of EETs and 20-HETE [11]. We electrically stimulated slices in order to evaluate the effect of neuron activation on the cPLA_2_α and IP_3_R2-dependent vascular regulatory pathways. Electrical stimulation resulted in astrocyte Ca^2+^ responses (Supporting Figure S2) and does not directly activate VSMC [38]. In our study, electrical stimulation caused dilation in both the naïve and U-46619 preconstricted arterioles while it had no vasomotor effect on IP_3_R2**^−^** ^/−^ or cPLA_2_α**^−^** ^/−^ arterioles. This implies that electrical activation of neurons (as compared to 1S,3R-ACPD activation of astrocytes) either increases the relative amount of a cPLA_2_α-dependent vasodilator or decreases the amount a of cPLA_2_α-dependent vasoconstrictor that is produced by activation of the astrocyte IP_3_R2. A mechanism for neuronal regulation of the astrocyte cPLA_2_α-dependent response was first suggested by the finding that NO inhibits the synthesis of EETS and 20-HETE [39]. Indeed, in rat retinas NO appears to be a determinant in the polarity of light-induced vascular responses with NO production favoring vasoconstriction because epoxygenanse activity (metabolizes arachidonic acid to EET, a vasodilator) is inhibited at lower NO concentrations than is Ω-hydroxylase (metabolizes arachidonic acid to 20-HETE, a vasoconstrictor) [11]. We found that inhibition of neuronal NOS with L-NPA pre-treatment prevented the electrical stimulation-induced dilation in cPLA_2_α^+/+^ slices. We hypothesize that electrical stimulation causes neuronal NO release which inhibits production of 20-HETE in the VSMC [40]. In contrast, when nNOS is inhibited by L-NPA the Ω-hydroxylase is no longer blocked by NO and production of 20-HETE causes arteriole constriction that opposes the dilating effects of EETs and PGs. In agreement with our results Liu et al. (2008) tested the effect of NOS expression on functional hyperemia in the whisker barrel cortex of rats and concluded that increased NO production following whisker stimulation may suppress 20-HETE synthesis [21]. Taken together, our data indicate that the vascular response to astrocyte Ca^2+^ signaling depends upon both the pre-existing tone of the arteriole and the particular signaling pathways that a stimulus triggers.

Our experiments also demonstrate that regulation of cerebral arteriole responses to vasoactive eicosanoids is dependent upon levels of tissue oxygenation. We used PGE_2_ to demonstrate that arteriole responses remained intact at the conclusion of each series of measurements. As expected, the response to PGE_2_ was independent of the IP_3_R2 or cPLA_2_α expression in the slice because PGE_2_ is a downstream metabolite of arachidonic acid. To our surprise, 10 µM PGE_2_ bath application caused arteriole constriction in the 95% O_2_ and dilation in the 20% O_2_ environment. This result is identical to the responses seen with activation of the mGluR and is consistent with a model in which the response to PGE_2_ is dependent on the metabolic and oxidative state of the region. In other studies, in rat brain slices, similar doses of PGE_2_ were described as an arteriole vasodilator [5], [9]. In one of these studies the slices were maintained in an ACSF solution with 2.8 mM glucose in contrast to the 20 mM glucose used in our ACSF [5]. This lower glucose concentration could favor glycolysis and thus dilation [9]. In the other study the response to PGE_2_ in high O_2_ was not evaluated while the low O_2_ response was identical to our result [9]. Because arteriole responses to PGE_2_ are largely determined by activation of the eicosanoid receptors of the VSMC and vascular endothelial cells we hypothesize that the regional metabolic state could alter the binding of PGE_2_ on eicosanoid receptors [41]. For example, in renal interlobular arterioles PGE_2_ causes constriction by binding the prostaglandin E type 3 (EP3) receptor [42] and in rat aortic rings concentrations of PGE_2_>1 µM cause constriction through activation of the thromboxane A_2_ receptor [43], [44]. We tested the possibility that constriction in 95% O_2_ was related to the high concentration of PGE_2_ used in the experiment by performing a dose response titration. In the high O_2_ environment 100 nM PGE_2_ still caused arteriole constriction and dilation was not observed at any concentration (not shown). Thus it is possible that the vascular responses to PGE_2_ are regulated by metabolic state in which high O_2_ favors binding to prostaglandin E receptors (EP) that cause constriction (EP1 and EP3) relative to those that cause dilation (EP2 and EP4). This hypothesis requires further testing because expression of thromboxane and EP receptors in the brain microvasculature has not been characterized and the dependence on oxidative state of specific prostaglandin binding on receptors has not been explored [45].

Our work is largely consistent with previous findings using pharmacological inhibition of PLA_2_s and arachidonic acid metabolic enzymes. This is in contrast to a study in which a cPLA_2_α-deficient mouse had a normal in vivo circulatory response to sensory stimulation [46]. It has been proposed that in this in vivo study the compensatory mechanisms may overcome the permanent genetic loss of cPLA_2_α and maintain normal circulatory responses [38]. Our work demonstrates that this is not the case since there is no evidence of compensation in the vascular responses of our knock-out mice in the slice preparation. It is also important to note that the kinetics of the vascular response in the slice model are significantly slower than those measured in vivo. Our results are consistent with previously published work using similar slice conditions. For example, Gordon and colleagues found that in high O_2_ concentration constriction was maximal 4 minutes after stimulation while dilation peaked 17 minutes after stimulation [9]. The slow kinetics of the slice model may be due to a number of factors. Vessel diameters are determined by the summation of constrictive and relaxing forces and the rates of change in diameter are likely due to the size of gradients in these forces. The lack of arteriole pressure in brain slice arterioles will decrease the dilation gradient and it is possible that contractile pressures are also reduced as the tissue of a slice can easily expand. Our results are also consistent with a pathway in which multiple sequential enzymatic steps are required to generate vasoactive compounds. It is possible that bath perfusion of the slices delays achieving the maximum concentration of arachidonic acid metabolites. Another possible reason for the difference between the in vivo and in slice models is the normal pH that we used to maintain slice health. The metabolic state of astrocytes impacts the polarity of vascular responses and regional acidosis appears to have a profound impact [9]. cPLA_2_α activity is sensitive to pH and it is possible that metabolic alterations that lower cellular pH could decrease cPLA_2_α activity within the astrocyte. Finally, it is likely that other regulatory pathways exist in vivo but not in slice. There are likely to be many other factors that influence cerebral vascular regulation.

The magnitude of the stimulated astrocyte Ca^2+^ response may also determine whether an arteriole constricts or dilates [38]. We found that the presence or absence of cPLA_2_α had no apparent effect on the Ca^2+^ response of astrocytes to 1S,3R-ACPD (Figure 7). Similarly the treatment of the slices with U-46619 did not cause any immediate Ca^2+^ response in the astrocytes nor did it alter the relative Ca^2+^ response of astrocytes in slices that were subsequently treated with 1S,3R-ACPD (not shown). This is consistent with an absence of thromboxane A_2_ receptors in perivascular astrocytes [47]. Indeed while addition of U-46619 alters the resting tone and Ca^2+^ responsiveness of the vascular smooth muscle cells of the arterioles [48] it appears to have little, if any effect upon the perivascular astrocytes. Therefore the changes in polarity of the arteriole response to 1S,3R-ACPD are unlikely to be due to changes in the concentration of Ca^2+^ within the astrocyte. In this model, astrocyte cPLA_2_α generates arachidonic acid and the responses of the vascular system to this arachidonic acid release are determined by its metabolism and other factors.

While other investigations have used bath application of U-46619 to achieve an equilibrium arteriole diameter in brain slices [10] we were not able to replicate this result. Bath application with 100 nM U-46619 caused progressive arteriole constriction that did not equilibrate. In preliminary work we found that higher concentrations of U-46619 caused arteriole occlusion and prevented subsequent responses to chemical dilators and constrictors. Application of lower concentrations of U-46619 slowed the constrictive response but did not achieve a stable arteriole diameter within the time frame of the experiments (not shown). In a model that continuously bath applies a dose of U-46619 that is ∼5 fold above the EC_50_ for thromboxane receptor occupancy it is not surprising that constriction progresses until the arteriole is completely constricted [49].

Neurons, smooth muscle, and endothelial cells also express cPLA_2_α [50]–[52] and because cPLA_2_α is globally deficient in the cPLA_2_α**^−^** ^/−^ mouse we must qualify our conclusions. While the results of this study are consistent with the model in which astrocyte cPLA_2_α is the generator of arachidonic acid mediators it remains possible that other cellular sources of cPLA_2_α are important in this signaling process. It will be necessary to create cell-line specific gene deletions to further test these possibilities.

We recognize that these experiments leave questions that can be answered by future investigations both in brain slices and *in vivo*. Our results indicate that activation of the mGluR triggers Ca^2+^ release through the IP_3_R2 receptor and that this increased Ca^2+^ allows cPLA_2_α to release arachidonic acid which is metabolized to vasoactive metabolites. Other forms of PLA_2_ can act synergistically with cPLA_2_α to amplify arachidonic acid release and lipid mediator generation [53] so while cPLA_2_α may be the first PLA_2_ activity in the signaling pathway others may also be necessary. Synaptic activity and the resting tone of the VSMC influence the magnitude and direction of arteriole responses to stimulation and their precise interactions with cPLA_2_ require further investigation. Importantly while cPLA_2_α blockade appears to be neuroprotective in excitotoxicity models the current results suggest that chemical inhibition of cPLA_2_α may significantly impair normal mechanisms of neurovascular regulation [54], [55].

## Methods

### Slice Preparation and Imaging

Brains were removed from P20–35 mice after decapitation. Coronal slices of the somatosensory cortex (300 µm thick) were cut on a Leica VT1200S vibrating tissue slicer (Leica Biosystems, Richmond, IL) equipped with a sapphire blade in ice-cold cutting saline (in mM): 135 N-methyl-D-glucamine chloride (NMDG), 1 KCl, 1.2 KH_2_PO_4_, 0.5 CaCl_2_, 1.5 MgCl_2_, 24.2 Choline Bicarbonate, 13 glucose, adjusted to pH 7.4 and oxygenated with 95% O_2/_5% CO_2_. Slices were then maintained in ACSF (in mM): 125 NaCl, 2.5 KCl, 1 NaH_2_PO_4_, 26.2 NaHCO_3_, 2.5 CaCl_2_, 1.3 MgCl_2_, 20 glucose (pH = 7.4), for at least 1 h at room temperature. For recording and imaging, slices were placed in a submerged chamber superfused with ACSF at a rate of 1–2 ml/min at 34°C. A 10 minute period of baseline recording preceded brain slice stimulation. For experiments with 1S,3R-ACPD, this drug was bath applied at indicated concentrations for 10 min followed by a 10 min washout period. Following this, the responsiveness of the chosen arteriole was evaluated by the addition of PGE_2_. In experiments with U-46619 was added to the ACSF after the initial 10 min stabilization period at the indicated concentrations and applied continuously throughout the experiments. For electrical stimulation, a concentric bipolar electrode was placed 200–300 µm lateral to the arteriole of interest. The stimulation protocol consisted of 100 Hz monophasic pulse trains of duration 200 msec, with an intertrain interval of 5 sec for a total duration of 4 min.

Cell structure within cortical slices was visualized through a 40X water immersion objective with gradient contrast optics using a fixed-stage upright microscope equipped with a Zeiss Pascal confocal system with Argon ion (488 nm), and HeNe (543 nm) lasers. Arterioles in each slice were identified by their characteristic size (inner diameter of 5–20 µm) and the presence of a vascular smooth muscle layer. We selected arterioles from cortical layers 2–5 that could be observed for a minimum length of 200 µm and then measured changes in the inner diameter. For each arteriole a baseline image was obtained and we established between 5–10 reference lines across the arteriole lumen. These reference lines were spaced at ∼5 µm intervals and were applied at the same axial location of the vessel for all subsequent radial measurements. At each time point one arteriole image was obtained and a second was obtained 30 seconds later. These images were digitally superimposed and radial measurements were taken from the resultant image. An investigator who was blinded to the experimental condition and genotype of the slice measured the arteriolar internal diameter for each time point.

After cutting, slices were incubated in ACSF saturated with 95% O_2_/5% CO_2_ for 60 minutes. They were transferred and maintained in ACSF saturated with either 95% O_2_/5% CO_2_ or 20% O_2_/5% CO_2_ depending upon the experiment. For low O_2_ experiments, slices were equilibrated in 20% O_2_ saturated ACSF for at least 40 min before experiments. The switch from high O_2_ to low O_2_ caused a small constriction of vessels (1.50±0.84%, P = 0.095, n = 17).

To block group I mGluRs, 100 nM JNJ (an antagonist of mGlu_1_) and 10 µM MPEP (an antagonist of mGlu_5_) were bath applied for 30 min before electrical stimulation. Blockade of mGluRs did not change vessel tone (dilation: 0.86±1.83%, P = 0.65, n = 9). JNJ and MPEP were dissolved in ethanol (final ethanol concentration: 0.02%).

To eliminate NO generated by neurons 10 µM L-NPA, a highly selective nNOS inhibitor, was bath applied for 60 min before electrical stimulation. L-NPA had little effect on vessel tone (constriction:1.46±1.93%, P = 0.47, n = 9). L-NPA was dissolved in water.

We used Rhod-2/AM as a cell-permeant indicator for Ca^2+^ imaging experiments. It was dissolved in DMSO together with the detergent Pluronic F-127 and then diluted with HEPES-ACSF (in mM) (125 NaCl, 2.5 KCl, 1 NaH_2_PO_4_, 25 HEPES, 2.5 CaCl_2_, 1.3 MgCl_2_, 20 glucose, adjusted pH to 7.4) to a final concentration of 10 µM (final DMSO concentration: 0.23%). Because the Ca^2+^ indicator Rhod-2/AM preferentially loads astrocytes [8] slices were incubated with Rhod-2/AM for 60–90 min at room temperature. Following loading, slices were maintained in ACSF. Astrocytes were selected for imaging on the basis of their uptake of Rhod-2 (or expression of EGFP), an ameboid-shaped cell body, a location in direct proximity to an arteriole, and the presence of a foot process in proximity to the arteriole. Rhod-2 was excited with 543 nm light while EGFP, marking a subpopulation of astrocytes of S100β-EGFP transgenic mice, was excited with 488 nm light. In this preparation 82±3% of EGFP astrocytes were loaded with red Rhod-2/AM (335 of cells, 17 slices, 8 mice). Rhod-2 images were acquired at 1.3 Hz/frame and signals were expressed as ΔF/F_0_ =  (F_t_-F_0_)/(F_0_-B_0_), where F_t_ is fluorescence intensity at any given time, F_0_ is the average fluorescence intensity in the baseline period and B_0_ is the average fluorescence intensity of background. Background values were taken from an adjacent region of interest (see Figure 7). For analysis of Ca^2+^ transients the 10–90% rise time and 90–10% decay times were calculated. Group data were expressed as mean ± SEM and compared by Student’s *t*-test.

### Mice

Mice were housed with a 12-hour diurnal light cycle and free access to food and water. All genetically altered mice used for experiments were produced by mating male and female mice that were heterozygous for the gene of interest. In these studies we used cPLA_2_α^+/+^ and cPLA_2_α**^−^** ^/−^ mice [54] that had been backcrossed on the BALB/C strain for >10 generations. Mice that were previously engineered to express a transgene for the EGFP protein under the control of the S100β promoter [22] were bred for greater than 6 generations with F1 progeny of BALB/c x cPLA_2_α**^−^** ^/−^ mating to create S100β-eGFP-cPLA_2_α^+/−^ mice. IP_3_R2**^−^** ^/−^ mice were originally supplied on the Swiss Webster background (Ju Chen, personal communication) and were mated with BALB/c WT mice to generate IP_3_R2^+/−^ mice [56]. All genotyping was performed from tail samples on mice between the ages of 8–12 days and were analyzed by PCR using specific primer pairs.

### Ethics

All studies were conducted with the approval of the Johns Hopkins University Animal Care and Use Committee under the protocol numbers MO07M135 and MO10M69. Performance of the studies was also in accordance with the guidelines of the National Institutes of Health and the National Research Council.

### Data Analysis

Changes in Rhod-2 fluorescence were analyzed using IGOR Pro 6 (Wavemetrics, Inc. Portland, OR) and expressed as cumulative probability histograms and mean values ± S.E.M. Arteriole diameters were measures using NIH Image J (NIH, Bethesda, MD) and expressed as mean values ± S.E.M. Data between groups were compared by Student’s *t*-test.

### Reagents

*1,1,1-trifluoro-6Z,9Z,12Z,15Z-heneicosatetraen-2-one (arachidonyl trifluoromethyl ketone, ATK)*, supplied as a solution in ethanol; final ethanol concentration: 0.0375%), PGE_2_ (supplied as a crystalline solid, dissolved in ethanol, final ethanol concentration: 0.35%), and U-46619 (supplied as a solution in methyl acetate, final methyl acetate concentration: 0.0007%) were purchased from Cayman Chemical Co. (Ann Arbor, MI). (1*S,*3*R*)*-*1-Aminocyclopentane-1,3-dicarboxylic acid (1S,3R-ACPD), Nω-propyl-l-arginine (L-NPA), JNJ 16259685, and 2-Methyl-6-(phenylethynyl)pyridine hydrochloride (MPEP) *were purchased from Tocris bioscience* (Ellisville, MO), and Rhod-2/AM was purchased from Invitrogen Corp. (Carlsbad, CA). All other chemicals were purchased from Sigma (St. Louis, MO). cPLA_2_α heterozygous mice bred into the BALB/C strain were used for all matings and were the gift of Joseph V. Bonventre (Brigham and Women’s Hospital, Boston, MA) [54]. IP_3_ type-2 receptor-deficient mice (IP_3_R2**^−^** ^/−^) were the gift of Ju Chen (University of California, San Diego, CA) [56]. Mice expressing the EGFP protein under the control of the S100β promoter were originally created in the laboratory of Legraverend (Institut de Génomique Fonctionnelle, Montpellier, France) [22] and were provided by Dwight E. Bergles (Johns Hopkins University, Baltimore, MD).

## Supporting Information

Figure S1 **Change in diameter of cortical arterioles upon sequential, combined exposure to U46619 and PGE_2_.** Cortical brain slices from IP_3_R2^+/+^ mice were at equilibrium with 95% O_2_, and treated with 100 nM U-46619 supplemented ACSF for 30 min. After 30 min ACSF was further supplemented with 10 µM PGE_2_ for an additional 10 min. n = 9 arterioles.
(TIF)

Figure S2 **Change in diameter of a single arteriole upon sequential, combined exposure to U46619 and 1S, 3R-ACPD.** Time is expressed in minutes with the t = 0 set at the initiation of U46619 and the initial diameter at t = −10 minutes. Bars indicate the time of bath application of 100 nM U46619 or 50 µM 1S, 3R-ACPD.
(TIF)

Figure S3 **Electrical stimulation evokes Ca^2+^ transient in astrocytes of a cortical slice derived from an S100β-EGFP mouse.** Slices were loaded with Rhod-2/AM and a concentric bipolar electrode was placed 200–300 µm from the region of interest. Rhod-2 fluorescence (red) in multiple astrocyte cell bodies that express EGFP (green) (circled in white; left panel) was measured after stimulation at 100 Hz for 200 ms (expanded black bar, right panel). The Ca^2+^ fluorescence signals of individual astrocytes are plotted. Scale bar: 20 µM.
(TIF)
