# Supplementary figures and images for: Astrocyte Inositol Triphosphate Receptor Type 2 and Cytosolic Phospholipase A2 Alpha Regulate Arteriole Responses in Mouse Neocortical Brain Slices
Source: PLoS One. 2012 Aug 2;7(8):e42194. doi: 10.1371/journal.pone.0042194 (PMC3410924; doi:10.1371/journal.pone.0042194)

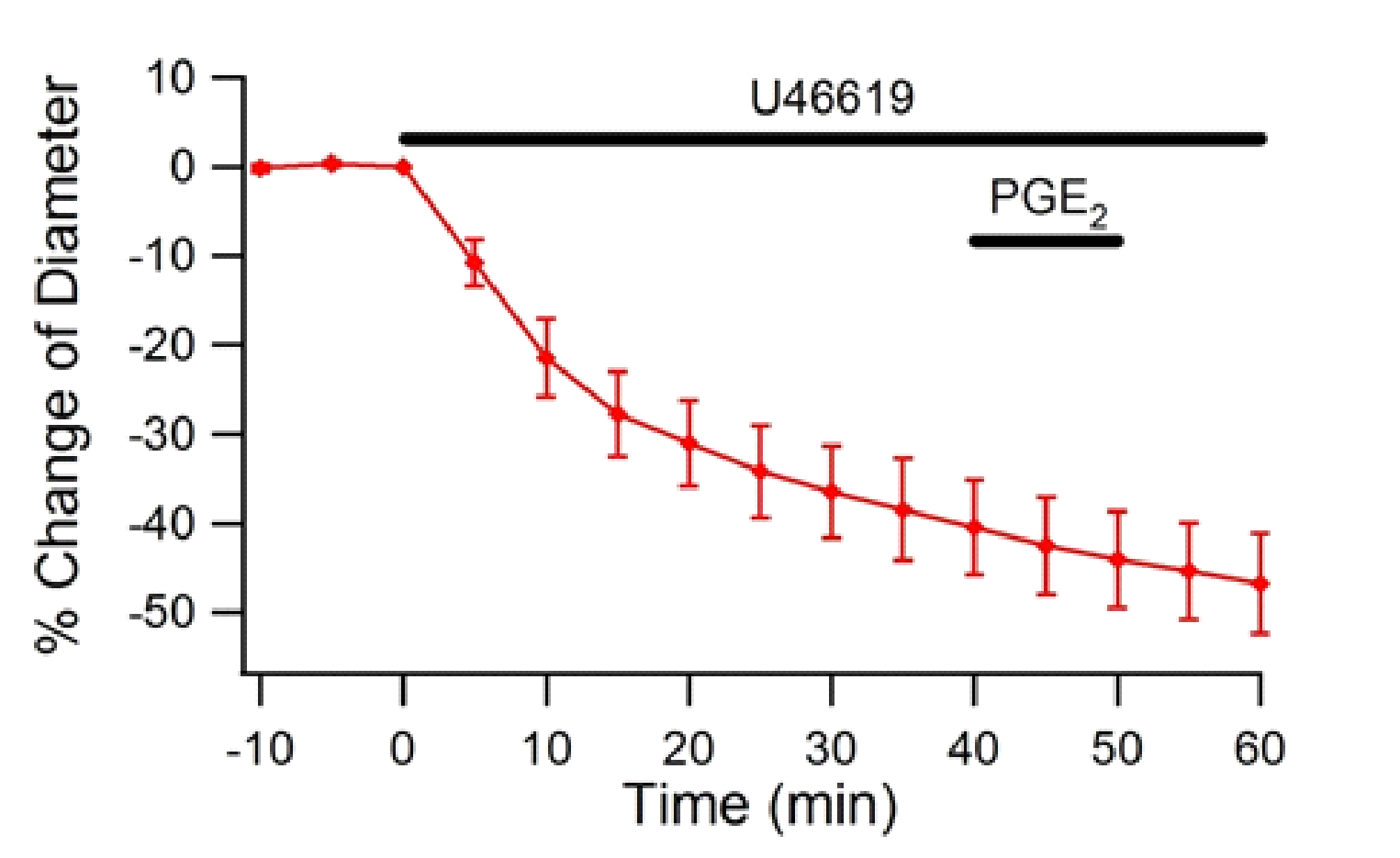

Supplement: Figure S1 — Change in diameter of cortical arterioles upon sequential, combined exposure to U46619 and PGE2. Cortical brain slices from IP3R2+/+ mice were at equilibrium with 95% O2, and treated with 100 nM U-46619 supplemented ACSF for 30 min. After 30 min ACSF was further supplemented with 10 µM PGE2 for an additional 10 min. n = 9 arterioles. (TIF) [file pone.0042194.s001.tif]

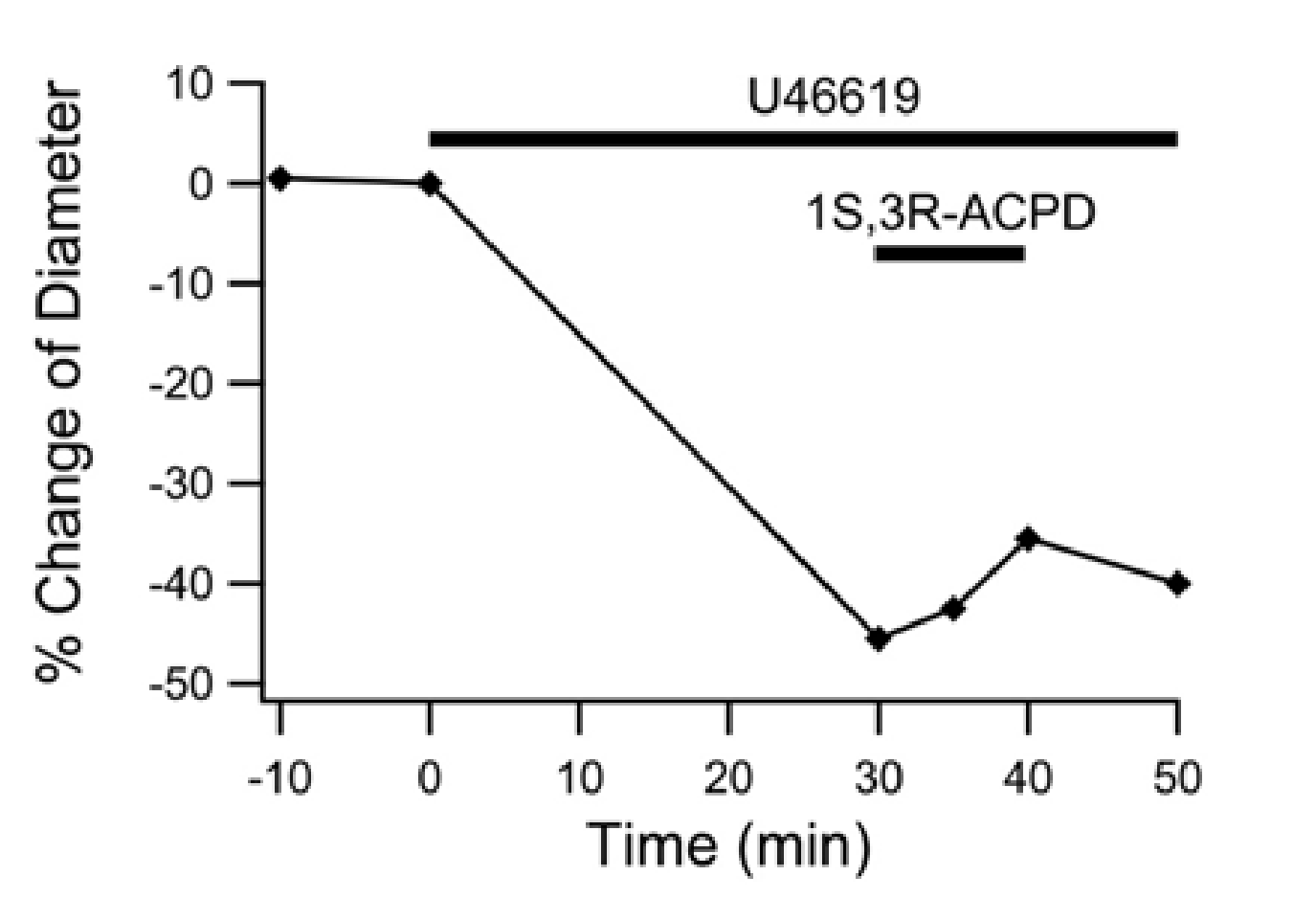

Supplement: Figure S2 — Change in diameter of a single arteriole upon sequential, combined exposure to U46619 and 1S, 3R-ACPD. Time is expressed in minutes with the t = 0 set at the initiation of U46619 and the initial diameter at t = −10 minutes. Bars indicate the time of bath application of 100 nM U46619 or 50 µM 1S, 3R-ACPD. (TIF) [file pone.0042194.s002.tif]

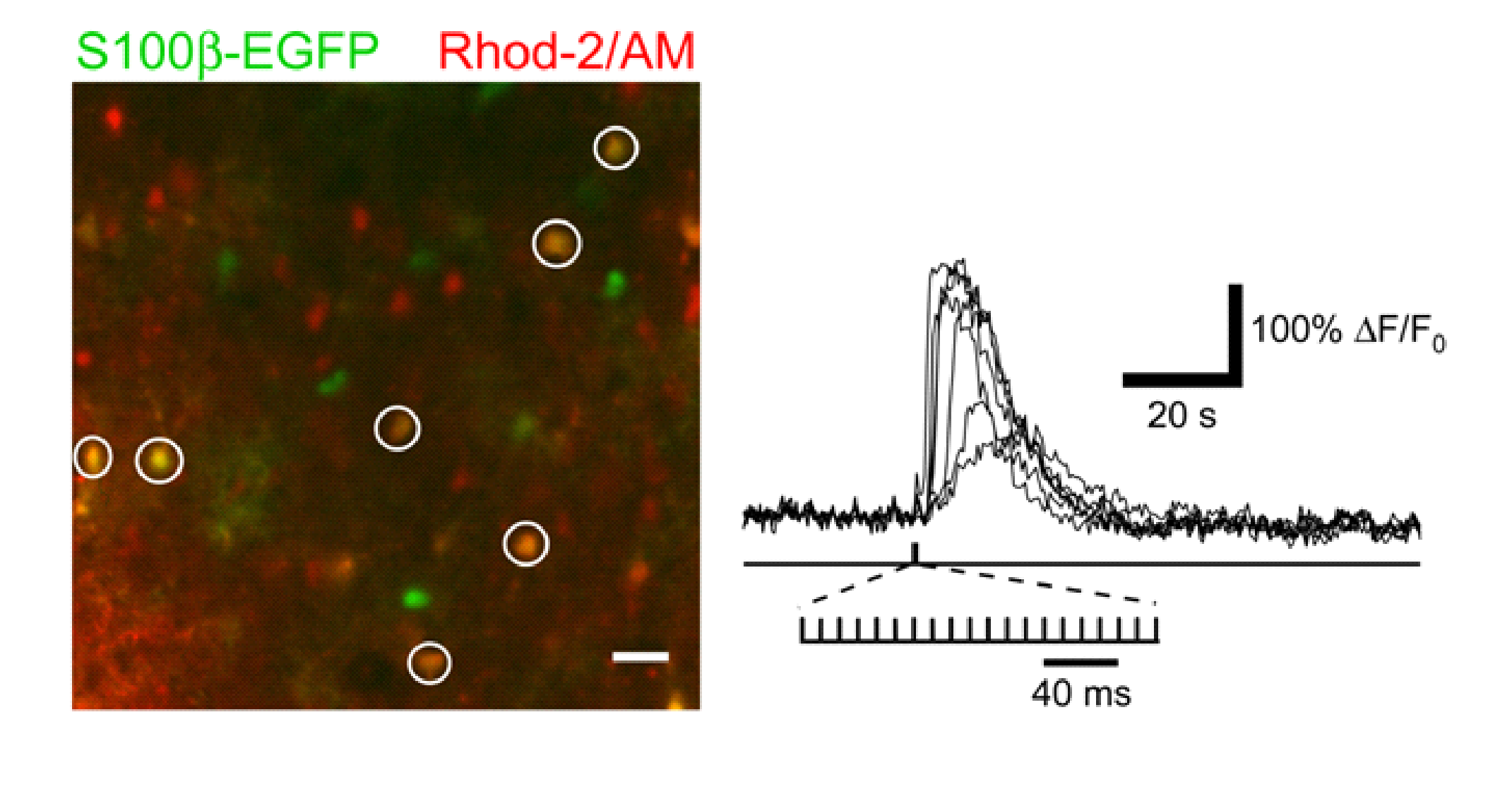

Supplement: Figure S3 — Electrical stimulation evokes Ca2+ transient in astrocytes of a cortical slice derived from an S100β-EGFP mouse. Slices were loaded with Rhod-2/AM and a concentric bipolar electrode was placed 200–300 µm from the region of interest. Rhod-2 fluorescence (red) in multiple astrocyte cell bodies that express EGFP (green) (circled in white; left panel) was measured after stimulation at 100 Hz for 200 ms (expanded black bar, right panel). The Ca2+ fluorescence signals of individual astrocytes are plotted. Scale bar: 20 µM. (TIF) [file pone.0042194.s003.tif]
